# Detection of *Micrococcus Luteus* Biofilm Formation in Microfluidic Environments by pH Measurement Using an Ion-Sensitive Field-Effect Transistor

**DOI:** 10.3390/s130202484

**Published:** 2013-02-18

**Authors:** Koji Matsuura, Yuka Asano, Akira Yamada, Keiji Naruse

**Affiliations:** 1 Research Core for Interdisciplinary Sciences, Okayama University, 3-1-1 Tsushima-naka, Kita-ku, Okayama 700-8530, Japan; E-Mail: dns18422@s.okadai.jp; 2 Department of Mechanical Systems Engineering, Faculty of Engineering, Hiroshima Institute of Technology, 2-1-1 Miyake, Saeki-ku, Hiroshima 731-5193, Japan; E-Mail: yamada@me.it-hiroshima.ac.jp; 3 Cardiovascular Physiology, Graduate School of Medicine, Dentistry and Pharmaceutical Sciences, Okayama University, 2-5-1 Shikata-cho, Kita-ku, Okayama 700-8558, Japan

**Keywords:** ion-sensing field-effect transistor, *Micrococcus luteus* biofilm, alkalinization, microfluidic channel

## Abstract

Biofilm formation in microfluidic channels is difficult to detect because sampling volumes are too small for conventional turbidity measurements. To detect biofilm formation, we used an ion-sensitive field-effect transistor (ISFET) measurement system to measure pH changes in small volumes of bacterial suspension. Cells of *Micrococcus luteus* (*M. luteus*) were cultured in polystyrene (PS) microtubes and polymethylmethacrylate (PMMA)-based microfluidic channels laminated with polyvinylidene chloride. In microtubes, concentrations of bacteria and pH in the suspension were analyzed by measuring turbidity and using an ISFET sensor, respectively. In microfluidic channels containing 20 μL of bacterial suspension, we measured pH changes using the ISFET sensor and monitored biofilm formation using a microscope. We detected acidification and alkalinization phases of *M. luteus* from the ISFET sensor signals in both microtubes and microfluidic channels. In the alkalinization phase, after 2 day culture, dense biofilm formation was observed at the bottom of the microfluidic channels. In this study, we used an ISFET sensor to detect biofilm formation in clinical and industrial microfluidic environments by detecting alkalinization of the culture medium.

## 1. Introduction

Bacterial biofilm formation often causes serious problems in the dental care field [1], artificial urinary tracts [2], and wastewater treatment systems [3]. Although basic technologies for the detection and control of biofilm formation in food processing plants have been reported [4], the underlying mechanism is not well understood and solutions to these problems are eagerly awaited. Microfluidic technologies [5,6] may be powerful tools for clarifying the mechanisms of biofilm formation. Although measurements of bacterial cell activity and growth in channels would be indispensable, few practical methods have been reported because of the required sample volume problem [7]. Conventional turbidity measurement techniques usually require 1 mL of sample solution for optical detection. Given that bacterial proliferation increases the pH of medium [8], precise measurement of pH changes induced by bacterial proliferation and/or metabolism would contribute to the detection of biofilm formation in microfluidic channels. Using this technology, problems induced by bacterial growth in the channels of artificial urinary tracts and industrial plants can be detected. Because bacterial metabolism produces basic compounds such as urea and arginine, these substances may also be indicators of bacterial growth-related problems [9].

Ion-sensitive field-effect transistor (ISFET) sensors require several microliters of solution to measure pH. ISFETs are metal-oxide-semiconductor field-effect transistors wherein the gate connection is separated from the chip in the form of a reference electrode inserted in an aqueous solution that is in contact with the gate oxide [10]. In the case of pH-sensitive field-effect transistors, the electrical signal generated depends on the surface potential of a gate insulator, which can be modified by the accumulation of charges at the insulator surface caused by pH variations occurring in the solution [11]. The tip diameter of the present ISFET is 0.5 mm, drastically smaller than that of a glass electrode, and thus reduces the quantity of solution required for measurement [12]. ISFETs have been applied to the culture medium of adherent mammalian cells [11–14], sea urchin embryos [15], and bacteria [8,16–18] in order to analyze metabolism of these organisms. Moreover, ISFETs have been adapted by fabricating microtanks from poly(dimethylsiloxane) [16,17]. This technology allows detection of bacterial proliferation over several minutes by monitoring pH variations. ISFETs have also been applied to measure pH in bacteria–dental cement interfaces. Mayanagi *et al.* suggested that conventional dental cements for filling restoration inhibited decrease in pH by bacterial acid production from sugar metabolism [18]. Furthermore, Ponsonnet *et al.* analyzed the pH of bulk medium containing bacteria and of a liquid phase that was in close contact with the surface of ISFET immersed in the culture medium [8].

In this study, we demonstrate that the ISFET sensor can be applied to detect biofilm formation in microfluidic channels using only 20–30 μL of bacterial suspension. This sensor could detect biofilm formation in 1 mL and 20 μL bacterial suspensions in microtubes and microfluidic channels, respectively. After 2 days of culture, bacterial growth was saturated and medium was alkalinized in 1 mL suspensions. Biofilm formation was visually confirmed at the bottom of microtubes. We could not observe many biofilms at the bottom of microfluidic channels during the acidification phase; however, during the subsequent alkalinization phase, dense biofilm formation was recognized by microscopic observations. These results indicate that mechanisms of cell metabolism and biofilm formation can be analyzed in microfluidic environments by detecting pH of bacterial suspensions. This technology is applicable to basic bacterial systems biology and to industrial plants such as food processing facilities.

## 2. Experimental Section

### 2.1. Bacterial Culture

**Figure 1.**
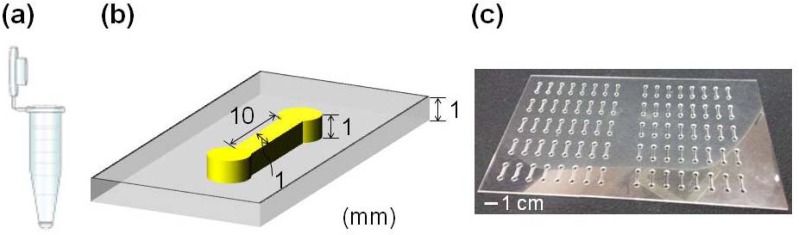
Bacterial culture systems used in this study. (**a**) Schematic view of PS microtubes and (**b**) microfluidic channels. (**c**) A microfluidic channel array.

Nutrient Broth (NB) was prepared using 0.8 g of Difco nutrient broth (Becton Dickinson and Company, Sparks, MD, USA), 0.1 g of yeast extract (Becton Dickinson and Company), and 0.5 g of casamino acids (Nihon Seiyaku, Tokyo, Japan) in 100 mL of distilled water. *Micrococcus luteus* (*M. luteus*; ATCC #9341, bio-safety level 1) cells were suspended in NB [19] and were cultured in 1.5 mL polystyrene (PS) microtubes (AS ONE, Osaka, Japan) as shown Figure 1(a) and in polymethylmethacrylate (PMMA)-based microfluidic channels laminated with polyvinylidene chloride (Saran F310 and F210; Asahi Kasei Chemical Industry Co. Ltd., Tokyo, Japan) at 30 °C. The microfluidic channels shown in Figure 1(b) were prepared according to a previous study [20]. We used a PMMA plate (thickness, 1 mm) containing 80 microfluidic channels [Figure 1(c)]. The volumes of medium in microtubes and microfluidic channels were 1 mL and approximately 20 μL, respectively.

It was not possible to culture the bacterial medium from microfluidic channels after pH and biofilm assessments were conducted. Culture experiments also ended with crystal violet staining of biofilms. Hence, pH and biofilm analyses can only be performed once. Therefore, we prepared microfluidic channel arrays to increase sample numbers. In future studies, this array may be used to screen drugs that suppress the formation of biofilms.

### 2.2. Evaluation of Bacterial Growth and Biofilm Formation

Bacterial suspensions were precipitated by centrifugation at 14,000 rpm (Centrifuge 5424, Eppendorf, Hamburg, Germany). After discarding the supernatant, bacterial pellets were resuspended in NB and optical density (OD) at 600 nm was recorded using a photometer (UV-1800; Shimadzu Co. Ltd., Kyoto, Japan). The cell concentration was adjusted according to turbidity (OD = 0.01, 0.02, 0.07, 0.1, 0.2, 0.3, and 0.5), and suspensions were poured into microtubes or microchannels. Bacteria were cultured for 2 days at 30 °C. Bacterial concentration in microtubes was evaluated by turbidity measurement using the photometer after 1 and 2 days. Increased turbidity of the suspensions indicated bacterial growth. According to McFarland turbidity standards [21] a linear relationship exists between OD and bacterial concentration [5 × 10^7^ × OD (CFU/mL)] when OD is below 2.

The biofilm of *M. luteus* in the microfluidic channel was stained with 0.01 g/mL crystal violet (Waldeck GmbH and Company, Münster, Germany), washed with distilled water three times, and observed using a microscope with a 4× objective lens (Nikon Co., Tokyo, Japan). The stained biofilm was photographed using a charge coupled device (Detect Co. Ltd., Tokyo, Japan), and images were analyzed using Image J software (National Institutes of Health, Bethesda, MD, USA). We created binary images from grayscale microscopic images, and the black area in the region of interest (ROI) of the binary image was regarded as the biofilm. The threshold intensity for black pixels was 80 after converting to 8-bit grayscale images. The percentage of black pixels in the binary image was calculated, and this percentage was considered as the amount of biofilm at the bottom of the microfluidic channel. In this study, we analyzed turbidity and pH of bacterial suspensions in microtubes and pH and biofilm formation in microfluidic channels by microscopic analyses. As it is not possible to measure turbidity in microfluidic channels, we evaluated pH and checked biofilm formation for the microfluidic bacterial culture.

### 2.3. Development of an ISFET Device for Measurement of Bacterial Proliferation

**Figure 2.**
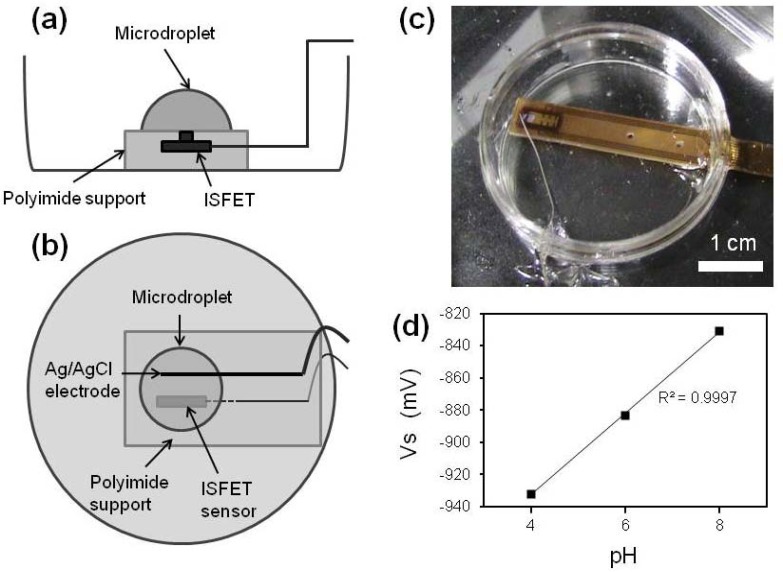
Detection device of the ISFET measurement system. (**a**) Side and (**b**) Top view of schematic presentation. (**c**) Photograph of the measurement system. (**d**) Linear relationship between pH and gate-source voltage (V_s_) of optimized buffers at pH 4, 6, and 8.

ISFET measurements were conducted according to previously described methods [13,14,22]. The gate region of ISFET used in this study was covered with a Ta_2_O_5_ membrane. The pH sensitivity at 25 °C was 50 mV/pH. To determine the pH of the culture medium, we prepared a measurement system comprising a well equipped ISFET (Figure 2). The sensor signal was sent to a PC (DOS/V) through an operating circuit (Automeasure Systems Co., Ltd., Okayama, Japan) and a 16-bit analog-to-digital converter (Contec, Osaka, Japan). The culture medium (10 μL) was dropped onto the sensitive region of the ISFET sensor (ISFETCOM, Saitama, Japan) [Figure 2(a)]. Before bacterial analyses, we applied optimized buffers of pH 4, 6, and 8 (Automeasure Systems Co. Ltd.) to calibrate the system. Figure 2(d) shows a linear response between pH of the solutions and the gate-source voltage (V_s_). The pH was measured at room temperature.

## 3. Results and Discussion

### 3.1. Turbidity and pH Changes in Microtubes

**Figure 3.**
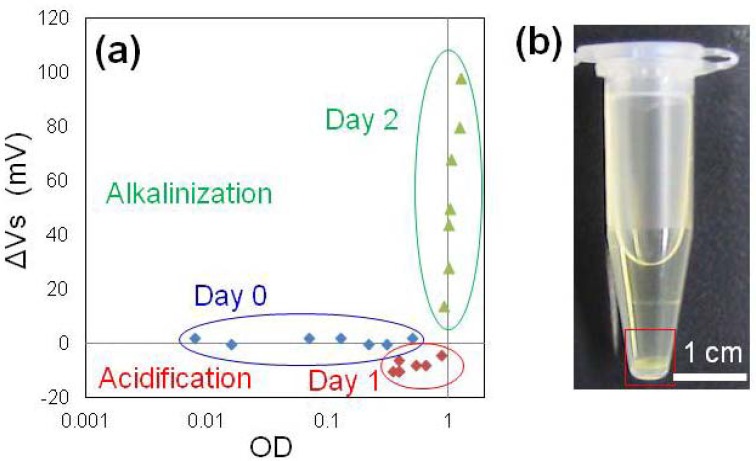
(**a**) Relationship between turbidity and pH measured by ISFET in medium containing *M. luteus*. Voltages in the vertical axis indicate the difference between the *M. luteus* suspension and the medium (ΔV_s_). Absorbance is shown in a log scale. (**b**) Biofilm formation in the PS microtube. A *M. luteus* biofilm was observed at the bottom of the microtube (red square).

Figure 3 shows the relationship between turbidity and V_s_ of bacterial suspensions in microtubes. After 1 day, the turbidity increased because of bacterial growth and slight acidification was observed by ISFET measurement, which corresponded to a previous report [8]. After 2 days, growth was saturated and alkalinization of medium occurred [Figure 3(a)], and biofilm formation was confirmed at the bottom of microtubes by visual examination [Figure 3(b)]. These results indicated that alkalinization occurred after the marked growth of the bacteria and that this phase could be suitable for biofilm detection because the temporal profile of alkalinization was similar to that of biofilm formation. Biofilm formation in PS microtubes could not be quantified as suggested in a previous study [7].

### 3.2. Biofilms and pH Changes in Microfluidic Channels

**Figure 4.**
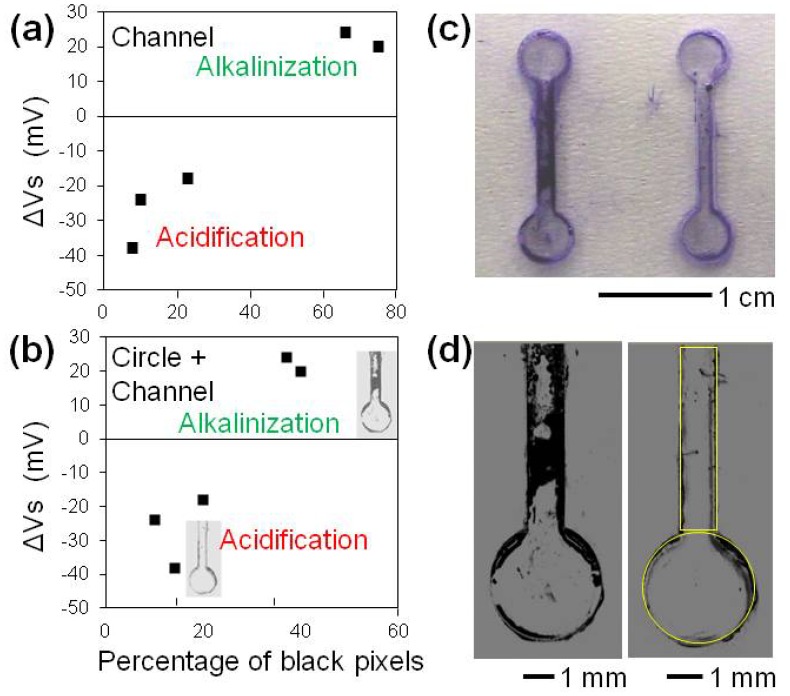
(**a**,**b**) Relationship between the percentage of stained biofilm coverage in the microfluidic channels and pH of the medium containing *M. luteus* measured using ISFET. Horizontal axes in (a) and (b) are percent coverage in channel regions and in circle and channel regions, respectively. Voltages in the vertical axes are the difference between those of *M. luteus* suspensions and medium (ΔV_s_). (**c**) Images of *M. luteus* biofilms formed in the microfluidic channels under alkalinized (left) and acidified (right) conditions. The biofilms were stained with crystal violet. The width of the microfluidic channel was 1 mm. (**d**) Grayscale microscopic images of the stained biofilms at the bottom of the microfluidic channels. The left and right images in (c) correspond to those in (d), respectively. Yellow rectangles and circles indicate ROIs in which the percent coverage of the biofilms was evaluated.

We analyzed biofilm formation at the bottom of microfluidic channels and pH change in *M. luteus* suspensions (Figure 4). Bacteria were cultured for 2 days in microfluidic channels. Biofilm formation was observed in some channels. The suspension in the microfluidic channel was removed using a micropipette, and the pH was measured using the ISFET sensor. We derived the percentage of biofilm covered area by processing grayscale microscopic images [Figure 4(d)].

Relationships between the percentage of stained biofilm coverage in the microfluidic channels and pH measured by ISFET are indicated in Figure 4(a,b). We observed some biofilms at the bottom of the microfluidic channels and slight acidification. In the alkalinization phase, dense biofilm formation was recognized by microscopic observation. This result is consistent with the analyses of 1 mL suspensions in microtubes [Figure 3(a)]. The ISFET sensor detected biofilm formation in the microfluidic channels using only 20–30 μL of bacterial suspension.

### 3.3. Discussion

**Figure 5.**
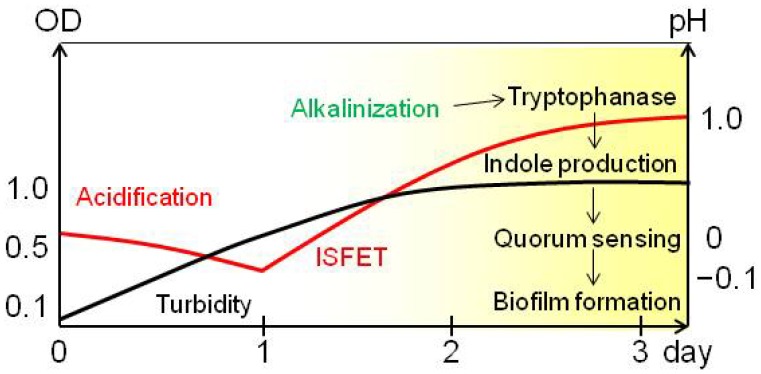
Schematic presentation of the mechanism of *M. luteus* biofilm formation. Biofilm formation was facilitated in the alkalinization phase. Changes in turbidity and pH measured using the ISFET sensor are shown with black and red lines, respectively. The yellow shadow indicates the phase of biofilm formation.

The temporal profile of acidification and alkalinization during bacterial biofilm formation is summarized in Figure 5. Based on pH measurements, *M. luteus* biofilm formation was facilitated after acidification and during alkalinization. This observation is consistent with that of previous papers [23,24]. Initially, organic acids are produced by metabolism of carbohydrates. These subsequently acidify the medium [9].

Under acidic conditions, bacteria that carry membrane-bound H^+^-gated urea transporters facilitate uptake of urea, which is metabolized by [9,25] urease dependent hydrolysis of urea to ammonia and carbon dioxide. Subsequently, secretion of ammonia from bacterial cells increases the pH of the medium following protonation to ammonium. Blankenhorn *et al.* reported that tryptophanase, which produces indole, was induced to a high level at pH 9 in *Escherichia coli*, becoming one of the most abundant proteins observed in two-dimensional gel electrophoresis [23]. Indole is proposed to be a signaling molecule of quorum sensing, a bacterial cooperation behavior sometimes concerning the biofilm formation [24]. Alkalinization, induction to increase tryptophanase, indole production, and quorum sensing are the possible steps leading to biofilm formation (Figure 5). These data indicate that alkalinization is the initial step in biofilm formation in *M. luteus*. The mechanism of biofilm formation in *M. luteus* is similar to that in *E. coli*. Moreover, we have shown that this mechanism can be investigated by integrating the ISFET sensor with analytical devices that detect tryptophanase, tryptophan, and/or indole. Therefore, by detecting pH during bacterial culture, the mechanism of cell metabolism and biofilm formation can be analyzed in microfluidic environments.

Changes in pH are dependent on the concentration of secreted bacterial metabolites. Although mechanistic details are debated, we discuss possible explanations for the variation in pH decreases between microtubes and microchannels. In particular, varying culture volumes and/or microfluidic environments affect bacterial metabolism, and thereby affects the secretion of metabolites. In future experiments we will quantitiate secreted substances in different culture environments to elucidate these mechanisms in more detail. Specifically, we will investigate kinetics of secretion by simultaneously quantifying pH using ISFET.

As previously reported, ISFET sensors can be installed in chip devices. Using microelectrodes (reference: calomel electrode), pH of 10 μL medium can be measured [26]. However, the electrode including the measurement system is too long (diameter, 2.8 mm and length, 150 mm) for application to microfluidic channels and/or devices. Because pH measurement using ISFET can be combined with confocal fluorescent microscopy for simultaneous analyses, the combined technology can be applied to research related to systems biological analyses for biofilm formation and bacterial communication mechanisms.

At present, there is no practical method for quantitative determination of biofilms in the food industry [4]. This measurement technology can be applied to food processing facilities. The measurement system for pH can be applied to the environment by sampling several microliters of polymeric substances or hot water.

## 4. Conclusions

We have demonstrated that the ISFET sensor can be used to detect biofilm formation in microfluidic channels and have determined the temporal profile of biofilm formation. The sensor indicated the degree of acidification and alkalinization of *M. luteus* by measuring the pH of medium in the microfluidic channels with internal volumes of 30 μL. The relationship between turbidity and pH of the medium indicated that the pH value could reflect both acidification and alkalinization phases, and marked alkalinization occurred after the saturation of OD values. Thus, the ISFET sensor provides some insight on secreted materials to induce pH change by culturing bacteria neighboring the sensor or in medium of microliter scale. We therefore propose that this measurement technology using the ISFET sensor could be a candidate for detecting bacterial biofilm formation in clinical and/or industrial microfluidic environments.
